# Diagnostics and prognostic evaluation in renal cell tumors: the German S3 guidelines recommendations

**DOI:** 10.1007/s00345-022-03972-x

**Published:** 2022-03-16

**Authors:** Kerstin Junker, Peter Hallscheidt, Heiko Wunderlich, Arndt Hartmann

**Affiliations:** 1 Department of Urology and Pediatric Urology, Saarland Medical Center, Saarland University, Kirrberger Str., 66421 Homburg, Germany; 2 Gemeinschaftspraxis für Radiologie und Nuklearmedizin, Worms, Germany; 3 Department of Urology and Pediatric Urology, St. Georg-Klinikum, Eisenach, Germany; 4 Institute of Pathology, University Erlangen-Nuremberg, Erlangen, Germany

**Keywords:** Renal cell carcinoma, S3 guideline, Prognosis, Tumor biopsy, Imaging

## Abstract

The German guidelines on renal cell carcinoma (RCC) have been developed at highest level of evidence based on systematic literature review. In this paper, we are presenting the current recommendations on diagnostics including preoperative imaging and imaging for stage evaluation as well as histopathological classification. The role of tumor biopsy is further discussed. In addition, different prognostic scores and the status of biomarkers in RCC are critically evaluated.

## Introduction

During the last 2 decades, therapeutic options in renal cell tumors changed not only in metastatic but also in organ confirmed disease due to a broader range of systemic therapies as well as surgical techniques. In addition, more and more small renal masses are detected, and therefore, surveillance strategies and ablative therapies are under discussion. Accordingly, exact diagnosis by imaging as well as histopathology and prognostic evaluation are necessary to select the optimal treatment for each individual patient. In addition, there is an urgent need for molecular biomarkers to further increase diagnostic accuracy including non-invasive markers as well as prognostic markers to individualize treatment. In this manuscript, we present the German highest level systematic literature review-based interdisciplinary guidelines concerning imaging, histopathological classification, renal tumor biopsy, prognostic evaluation, and biomarkers for renal cell tumors (Leitlinienprogramm Onkologie (Deutsche Krebsgesellschaft, Deutsche Krebshilfe, AWMF): Diagnostik, Therapie und Nachsorge des Nierenzellkarzinoms, Langversion 2.0,2020, AWMF Registernummer: 043/017OL, https://www.leitlinienprogramm-onkologie.de/leitlinien/Nierenzellkarzinom) [1].

## Methods

The methodological approach is described in detail in the guidelines [1]. Briefly, evidence grade is based on the Scottish Intercollegiate Guidelines Network (SIGN) system. Three grades of recommendation have been used (A: strong recommendation; B: recommendation; 0: recommendation not defined). Four categories were used to define grade of consensus (strong consensus: > 95%; consensus: > 75%-95%; consensus by majority: 50–75%; dissent: < 50% agreement). If systematic search was not performed, recommendations are based on expert consensus.

### Diagnostics: imaging

With the increasing number of incidentally detected renal cell carcinomas (RCC), the average size is decreasing continuously. The differential diagnosis of smaller lesions is difficult, as typical signs such as cava thrombus, necrosis, or metastasis are missing.

High-resolution imaging in CT and MRI can delineate even small and chromophobe carcinomas [2]. Staging accuracy of small RCC is similar in MRT and CT with staging accuracy between 0.78 and 0.87 [3]. CT is used routinely for small carcinomas [4], whereas tumor with caval thrombus should be staged with MRI [5] (Table 1).

**Table 1:** Evidence-based recommendations for diagnostic imaging

Evidence-based recommendation	Level of evidence (LoE)	Grade of recommendation	Consensus
For preoperative workup for local staging and for planning of nephron sparing surgery of renal cell carcinoma a triphasic CT has to be performed: unenhanced CT scan from the dome of the liver to the symphysis, in the early arterial phase from the dome of the liver to the lower pole of the kidneys in a parenchymal phase from the dome of the liver to the symphysi	1 +	A	Strong
Patients with renal cell carcinoma and suspected caval thrombus or venous infiltration should undergo MRI of the abdomen as a primary diagnostic modality. The MR should be performed according to a standard protocol	1 +	B	Strong

The CT scan should include an unenhanced spiral of the complete abdomen and a spiral in the early arterial phase of renal perfusion of the upper abdomen and a delayed scan of the complete abdomen. For the CT, a reconstructed slice thickness of 2 mm should be used. An enhanced scan of the thorax in a venous phase can be added for staging. Especially for planning of nephron sparing surgery, high-resolution 3D reconstructions are mandatory. CT has a good accuracy in the evaluation of infiltration into the perirenal fat [6], but has a limited accuracy in the evaluation of intrarenal infiltration [7].

MRI as diagnostic modality is recommended in case of allergic reactions to iodine containing contrast media (CT) or suspected caval thrombus. The complete abdomen including the atrium and the lower pole of the kidneys should be scanned. The scans should include axial T2w images, axial enhanced T1w images and a coronal multiphase acquisition with an unenhanced scan, an early arterial phase, and a parenchymal phase. In case of urogenital bleeding a delayed scan for complete urothelium including bladder should be added. Especially, the high-resolution T2w images allow a delineation of the tumor thrombus [4].

Sensitivity and specificity of caval thrombus evaluation are 1.0 and 0.83 for MRI [5].

For grading, RCC diffusion and perfusion imaging seem to be a promising tool [8–10]. As imaging can hardly differentiate between histologic subtypes, despite of classic AMl, additional biopsy might be useful for therapy planning.

Although prospective trials provide high level of evidence, more data are desirable to corroborate the recommendations.

### Imaging for evaluation of metastasis

In patients with tumor size of 3 cm and higher, unenhanced and enhanced thin slice CT (2 mm) of the thorax should be performed, because the risk of metastasis is increasing. CT has a much higher sensitivity and specificity to detect lung metastases than conventional chest X-ray as CT allows to detect small calcifications and fat in pulmonary lesions, and can therefore differentiate the pulmonary lesions [4, 11–13]. For detection of abdominal lesions, MRI and CT have similar detection rates. If brain metastases are suspected, MRI should be used due to its better capability to detect metastases and edema in the brain (Table 2).

**Table 2:** Expert consensus-based recommendations for evaluation of metastasis by imaging

Consensus-based recommendation	Grade of consensus
In asymptomatic patients with malignant tumors exceeding 3 cm, an enhanced CT of the thorax should be performed	Consensus
In case of suspected bone lesions, imaging has to be performed preferably by whole body CT (low dose) or MRI and not by scintigraphy	Consensus
In case of suspected brain lesions, an enhanced MR scan of the scull/brain has to be performed	Strong

### Biopsy

In cases of uncertain renal lesions, it would be helpful to perform biopsies for histopathological evaluation, especially in patients who are candidates for active surveillance or renal tumor ablation (Table 3). Volpe et al. described a 16% reduction of surgery [14]. However, there is the possibility of false-negative results.

**Table 3:** Expert consensus-based recommendations for renal tumor biopsy

Consensus-based recommendation	Grade of consensus
Biopsy of uncertain lesions of the kidney should be performed only if it impacts clinical management	Consensus
Biopsy is recommended before renal tumor ablation	Strong
Biopsy of cystic renal lesions should not be performed	Strong
Renal tumor biopsy or biopsy of metastases is recommended in patients with primary metastatic disease before systemic therapy if histopathological evaluation was not yet performed	Strong
Renal tumor biopsy can be offered before cytoreductive nephrectomy in metastatic patients	Consensus

Under local anaesthetics, percutaneous sampling can be performed as core biopsy or fine needle aspiration, alone or in combination, US or CT-guided. Fine-needle aspiration shows lower diagnostic yield and accuracy [14, 15], which can be improved by adding 18 Gauge core biopsy [16–18].

Systematic reviews reported a comparatively high diagnostic yield, sensitivity, and specificity for the diagnosis of malignancy (99.1% and 99.7%) when using core biopsy [14, 19, 20].

In tumors > 4 cm, peripheral ultrasound guided biopsies are recommended to avoid sampling of central areas with tumor necrosis [21].

An issue of renal lesion biopsy emphasizes the problem of managing negative biopsy results (0–22.6%). Repeat biopsy is diagnostic in most patients (83–100%) [22, 23]. Therefore, an indeterminate or negative biopsy result but suspicious imaging findings should prompt a repeat biopsy or be interpreted as RCC if repeated biopsy is impossible.

Core biopsy of cystic renal lesions has a lower diagnostic yield and accuracy compared to solid lesions and is therefore not recommended [24].

The morbidity of percutaneous biopsy is low [14, 19]. Tumor cell seeding along the needle tract is unlikely. In a recent pooled analysis, spontaneously resolving and clinically insignificant subcapsular perinephric hematoma was reported in 4.3% of biopsies [25].

### Histopathological classification

The recommendations are based on the consensus conference and the most recent guidelines [26–28] (Table 4).

**Table 4:** Expert consensus-based recommendations for histopathology

Consensus-based recommendation	Grade of consensus
The histological type of renal cell carcinoma should be defined according to the recent WHO classification The tumor types recommended by the Vancouver Classification of Renal Cell Carcinoma of the International Society of Urological Pathology (ISUP) should be diagnosed The diagnosis of the following new tumor types is recommended: Tubulocystic renal cell carcinoma Acquired cystic disease-associated renal cell carcinoma Clear cell papillary renal cell carcinoma MiT-family translocation renal cell carcinoma Hereditary leiomyomatosis and renal cell carcinoma-associated renal cell carcinoma	Strong
The most recent TNM classification should be used. The tumor grade should be diagnosed in clear cell and papillary renal cell carcinoma according to the WHO-ISUP grading. In addition, the proportion of tumor necrosis should be given	Strong
Chromophobe renal cell carcinomas should not be graded	Strong
The papillary renal cell carcinoma should be diagnosed in two different types (Type 1 and Type 2)	Strong
A sarcomatoid and/or rhabdoid differentiation should be mentioned	Strong

The WHO Classification of 2004 presented a comprehensive histopathological classification of RCC, which were revised in 2013 by the International Society of Urological Pathology (ISUP) in the Vancouver Classification [28]. In the consensus conference of the S3-Guidelines the diagnosis of the following new entities was recommended: Tubulocystic RCC, Acquired cystic disease-associated RCC, Clear cell papillary RCC, MiT-family translocation RCC with Xp11 translocation or t(16; 11) translocation, Hereditary leiomyomatosis, and renal cell carcinoma-associated RCC. These entities were also recommended by the WHO classification of 2016.

#### Renal cell tumors

- Papillary adenoma
- Oncocytoma
- Clear cell RCC
- Multilocular cystic renal neoplasm of low malignant potential
- Papillary RCC
- Chromophobe RCC
- Collecting duct carcinoma
- Renal medullary carcinoma
- MiT-family translocation RCC
  ○ Xp11 translocation RCC
  ○ t(16; 11) RCC
- Succinate dehydrogenase-deficient RCC
- Mucinous tubular and spindle cell RCC
- Tubulocystic RCC
- Acquired cystic disease-associated RCC
- Clear cell papillary RCC
- Hereditary leiomyomatosis and renal cell carcinoma-associated RCC
- Unclassified RCC.

#### Metanephric tumors

- Metanephric adenoma
- Metanephric adenofibroma
- Metanephric stromal tumors.

#### Nephroblastic tumors

- Nephroblastoma
- Cystic partially differentiated nephroblastoma
- Pediatric cystic nephroma.

#### Mesenchymal tumors occurring mainly in children

- Clear cell sarcoma
- Rhabdoid tumor
- Congenital mesoblastic nephroma
- Ossifying renal tumor of infancy.

#### Mesenchymal tumors occurring mainly in adults

- Angiomyolipoma
- Epitheloid angiomyolipoma
- Leiomyoma
- Hemangioma
- Juxtaglomerular cell tumor
- Renomedullary interstitial cell tumor
- Schwannoma
- Solitary fibrous tumor
- Neuroectodermal tumor
- Synovial sarcoma
- Leiomyosarcoma
- Angiosarcoma
- Rhabdomyosarcoma.

#### Mixed epithelial and stromal tumors

- Adult cystic nephroma/mixed epithelial stromal tumor (MEST).

#### Neuroendocrine tumors

- Low-grade neuroendocrine tumor
- High-grade neuroendocrine tumor/neuroendocrine carcinoma
- Neuroblastoma
- Pheochromocytoma.

#### Hematopoietic and lymphoid tumors

- Lymphoma
- Leukemia
- Plasmacytoma.

#### Germ cell tumors

##### Metastatic renal cell carcinoma

In the consensus meeting, the classical histopathological parameters and the grading system for RCC were discussed. It was recommended that the tumor grade of RCC should be given according to the WHO-ISUP grading system. There is a clear correlation of the grade with the prognosis in clear cell and papillary RCC. Papillary RCC should be separated in two types (Type 1 with low grade and basophilic cytoplasm and Type 2 with high grade and eosinophilic cytoplasm). Papillary RCC Type 1 has an excellent prognosis. Furthermore, it was recommended that chromophobe RCC should not be graded. Other histological features were discussed. A sarcomatoid and rhabdoid differentiation should be mentioned in the histopathological report, because it is clearly associated with a poorer prognosis. The proportion of necrosis is also associated with a poorer prognosis and should be given.

The grading of chromophobe RCC has to be improved. The new grading systems proposed by Paner et al., Avulova et al., and Ohashi et al. are based on the pattern of the tumor and show that necrosis and sarcomatoid dedifferentiation are the most important factors for an adverse outcome [29–31]. Therefore, this should be mentioned in every report, which is also stated in the guideline. However, prospective data are still lacking. Thus, the grading is still not recommended by the WHO.

The data for microvascular invasion in lymph or blood vessels as a poor prognostic factor are not sufficient.

A new edition of the TNM classification is available since 2017 [32].

### Prognostic scores

TNM staging and grading still represent the most important characteristics for prognostic evaluation. However, these parameters are not sufficient to evaluate the individual prognosis in a given patient. Therefore, several prognostic models have been developed to predict outcome at different time points of disease and therefore select patients for different therapeutic options. These models should improve the prognostic accuracy compared to standard TNM stage and grade (Table 5).

Although some preoperative nomograms exist, majority of models have been developed for postoperative evaluation. The first aim is to evaluate the risk of progression/metastasis and survival in local disease. The following nomograms have been created: UISS (UCLA Integrated Staging Aystem)-model [33], Karakiewicz-nomograms [34, 35], SSIGN-score [36], Leibovich-score [37], Kattan-nomogram [38], Sorbellini-nomogram [39], and papillary nomogram [40]. Some of these nomograms are developed for clear cell RCCs only; others did not differentiate histological subtypes. Most of them are validated in independent patient cohorts (Table 5). The second aim is to predict the outcome of metastatic patients treated with systemic therapy. The Motzer or MSKCC-score is the first and mostly used nomogram developed for patient cohorts treated with interferon [41]. However, it is still used in the tyrosine kinase inhibitors era, too. Additional nomograms include the IMDC or Heng-score [42], the International Kidney Cancer Working Group-Modell (IKCWG)-model [43], the Cleveland Clinic Foundation-Modell (CCF)-model [44], the French model [45], the Sunitinib model [46], and the Leibovich Score before immune therapy [47].

**Table 5:** Consensus- and evidence-based statements for using prognostic scores

Consensus-based statement	Grade of recommendation	Grade of consensus
Prognostic factors include performance status, occurrence of metastasis depending on time point and localization, symptoms, haematologic parameters (Hb value, number of thrombocytes and neutrophils) as well as LDH	Expert consensus	Consensus

Evidence-based statement	Level of evidence	Grade of consensus
Validated multivariable nomograms are available for distinct time points of disease and treatment. These models have a higher accuracy than single parameters	2 + +	Strong

Consensus-based statement	Grade of recommendation	Grade of consensus
Multivariable nomograms can be used for counselling of patients with RCC. Accuracy and validation data have to be considered	Expert consensus	Strong

Currently, Motzer score and the IMDC-nomogram are most frequently used models to categorize patients in risk groups and thereof to predict outcome in clinical trials and practice.

### Biomarkers

Diagnostic biomarkers could improve early detection and differential diagnosis in addition to histopathological evaluation, especially in small renal masses. In addition, there is an urgent need in prognostic biomarkers to define individual outcome of RCC patients and, therefore, to select the most appropriate therapy.

During the last years, several biomarkers on different molecular levels (DNA, RNA, and proteins) have been published that are correlated with metastasis and survival of RCC patients [48–51]. Some of these markers have been analyzed in comparison to existing clinical prognostic parameters or have been incorporated in prognostic scores, and improved prognostic accuracy [52–54]. Due to the lack of independent prospective validation, none of these markers has been introduced in clinical routine (Table 6).

**Table 6:** Consensus-based statement for biomarker use

Consensus-based statement	Grade of consensus
The evidence to use biomarkers for prognostic evaluation is currently too low	Strong

However, it is likely that new prognostic markers will be identified from complex high-throughput analyses on several molecular levels in parallel considering clinical course [55–58].

Currently, several molecular signatures alone or integrated into clinical models have been published which are partially validated in independent cohorts. Almost all have a superior accuracy to predict individual patient outcome compared to clinically based nomograms [57, 59–62].

## Data Availability

Not applicable.
